# Prolactin in Polycystic Ovary Syndrome: Metabolic Effects and Therapeutic Prospects

**DOI:** 10.3390/life13112124

**Published:** 2023-10-26

**Authors:** Lara Mastnak, Rok Herman, Simona Ferjan, Andrej Janež, Mojca Jensterle

**Affiliations:** 1 Faculty of Medicine, University of Ljubljana, 1000 Ljubljana, Slovenia; 2 Department of Endocrinology, Diabetes and Metabolic Diseases, University Medical Center Ljubljana, 1000 Ljubljana, Slovenia; 3 Department of Internal Medicine, Faculty of Medicine, University of Ljubljana, 1000 Ljubljana, Slovenia

**Keywords:** polycystic ovary syndrome, prolactin, metabolic homeostasis, cardiometabolic markers, dopamine agonist

## Abstract

Polycystic ovary syndrome (PCOS) is the most prevalent endocrine and metabolic disorder in premenopausal women, characterized by hyperandrogenism, ovulatory dysfunction, and polycystic ovaries. Patients frequently present comorbidities, including obesity, insulin resistance, and impaired glucose and lipid metabolism. The diverse clinical presentation may mimic various endocrine disorders, making the diagnosis challenging in some clinical circumstances. Prolactin (PRL) is a recommended biomarker in the initial diagnostic workup to rule out hyperprolactinemia (HPRL). The traditional role of PRL is linked to lactation and the reproductive system. Recent research highlights PRL’s emerging role in metabolic homeostasis. PRL influences metabolism directly by interacting with the pancreas, liver, hypothalamus, and adipose tissue. Its influence on an individual’s metabolism is intricately tied to its serum concentration. While deficient and very high levels of PRL can negatively affect metabolism, intermediate–normal to moderately high levels may promote metabolic health. In women with PCOS, PRL levels may be altered. Research results on different aspects of the relationship between PCOS and the impact of various levels of PRL on metabolic homeostasis are limited and inconsistent. In this narrative literature review, we comprehensively examined data on serum PRL levels in PCOS patients. We investigated the correlation between a favorable metabolic profile and serum PRL levels in this population. Furthermore, we explored the concept of beneficial PRL effects on metabolism and discussed the potential therapeutic application of dopamine agonists in PCOS treatment. Lastly, we emphasized several promising avenues for future research in this field.

## 1. Introduction

Polycystic ovary syndrome (PCOS) is a multisystem, endocrinological, reproductive, and metabolic disorder [1,2,3]. Despite its most common clinical presentation, first described in 1935, with features of menstrual disturbances, hirsutism, and polycystic ovaries, it is nowadays recognized as a complex condition with multiple components, heterogeneous pathophysiology, and a polygenic basis [4,5].

### 1.1. PCOS Overview: Prevalence and Diagnostic Criteria

PCOS is the most prevalent endocrinopathy in the premenopausal phase, affecting women across their lifespan, from adolescence to postmenopause [6,7]. The global prevalence of the syndrome falls within the range of 10% to 13% and is experiencing exponential growth worldwide without specific regional or demographic limitations [6,8]. According to the 2023 International Evidence-based Guideline Criteria [6], at least two of the three following criteria are needed to confirm the diagnosis: clinical or biochemical hyperandrogenism (HA), ovulatory dysfunction (OD), and polycystic ovarian morphology (PCOM) on ultrasound. Alternatively, anti-Müllerian hormone (AMH) can be used in adult patients instead of ultrasound to diagnose PCOM [6]. Hyperandrogenemia is the primary biochemical indicator of PCOS and is prevalent in 75 to 90% of PCOS patients with oligomenorrhea. The degree of androgen elevation frequently corresponds to the severity of the phenotype. Excessive androgen production contributes to hirsutism, acne, and male-pattern alopecia, which are hallmarks of clinical HA [9]. OD clinically presents with irregular menstrual cycles. Approximately 80% of women with PCOS experience OD [10]. PCOM is an ultrasonographic observation that arises due to disruptions in follicular development, resulting in the presence of arrested follicles [11]. The criteria for diagnosing PCOM are based on the presence of an excess of follicles (≥20 follicles measuring 2–9 mm across the entire ovary or ≥10 in a specific section if using older ultrasound equipment) and/or an elevated ovarian volume (≥10 mL) [6]. Based on these diagnostic criteria, the syndrome can be categorized into four distinctive phenotypes: phenotype A (HA + OD + PCOM), phenotype B (HA + OD), phenotype C (HA + PCOM), and phenotype D (OD + PCOM). Phenotypes A and B, often termed “classic” PCOS, typically manifest with obesity, and patients exhibit more irregular menstrual patterns and more severe hirsutism. They are more prone to insulin resistance (IR), dyslipidemia, and hepatic steatosis and have a higher risk of metabolic syndrome (MS) compared to ovulatory or non-hyperandrogenic phenotypes (C and D) [10,12].

### 1.2. PCOS and Metabolic Implications: The Vicious Circle

PCOS presents with a variety of metabolic implications such as obesity, metabolic syndrome (MS), hyperinsulinemia, IR, and dyslipidemia [2,13,14] and thus presents an increased risk for type 2 diabetes mellitus (DM2) and cardiovascular disease [15]. Increased insulin levels and IR significantly contribute to the pathophysiology of PCOS and worsen the severity and complications of the syndrome [9,13,16]. 

Insulin can act synergistically with luteinizing hormone (LH) as a co-gonadotrophin within ovarian theca cells by enhancing the production of androgens. Moreover, insulin mediates follicular development, promoting the arrest of follicle development in the setting of hyperinsulinemia. Synthesis of sex hormone-binding globulin (SHBG), a key circulatory protein that regulates testosterone levels, is decreased by insulin. Therefore, lower SHBG levels lead to higher levels of free testosterone [9,13,16].

Women with PCOS consistently show an increased risk of subclinical atherosclerosis, as measured by various methods. The translation of subclinical atherosclerosis into higher rates of peripheral arterial disease and major cardiovascular events remains unclear. There are uncertainties regarding whether cardiovascular health is compromised in all patients or only specific subtypes, particularly those with obesity, and whether cardiovascular risk factors in reproductive age persist and lead to cardiovascular events and elevated mortality post-menopause [17]. 

### 1.3. PCOS: Diagnosis of Exclusion

PCOS can sometimes resemble other disorders [18,19]. Due to this possibility of overlap, PCOS is considered a diagnosis of exclusion. Biochemical and clinical assessments should rule out conditions such as thyroid dysfunction and hyperprolactinemia (HPRL), non-classic congenital adrenal hyperplasia (CAH), Cushing’s syndrome, hypogonadotropic hypogonadism, or virilizing tumors [2,6]. Prolactin (PRL) serves as a biomarker for the exclusion of HPRL, which can manifest with oligomenorrhea, amenorrhea, infertility, and mild hirsutism [20,21]. However, it has been reported that women diagnosed with PCOS might also concurrently present with HPRL [22]. 

### 1.4. Prolactin

PRL, a hormone produced by the pituitary gland, is primarily known for its role in stimulating the proliferation and differentiation of mammary cells necessary for lactation [23]. In addition, PRL has been associated with various functions, such as the regulation of the immune and reproductive systems [24]. Recently, its potential metabolic functions have come into focus [25,26,27]. 

It is reported that the role of PRL in metabolism highly depends on its serum concentrations. Low and very high levels can negatively impact metabolism, leading to obesity, MS, and disorders in glycemic and lipid profiles. On the other hand, medium and moderately high levels of PRL may promote metabolic homeostasis in physiological and pathological metabolic challenges [26,27]. Women with PCOS exhibit a broad range of PRL levels, ranging from higher than, equal to, or even lower than women without the syndrome [5,22].

### 1.5. Aim of the Review

In this narrative literature review, we first established the impact of PRL on metabolism, providing a foundation for our subsequent comprehensive exploration of its relationship with PCOS and its links to metabolic risk factors in affected individuals. Furthermore, we investigated whether an increase in PRL levels could be a part of an adaptive response to a range of metabolic challenges stemming from both normal physiological processes and pathological conditions. Then, we addressed the potential efficacy of pharmacological interventions using dopamine agonists that could modify PRL levels and their effects on the reproductive and metabolic health of women with PCOS. Lastly, we emphasized several knowledge gaps and promising avenues for future research in this field. 

We searched PubMed and Google Scholar databases using search terms “prolactin” in conjunction with “physiology”, “PCOS”, “polycystic ovary syndrome”, “metabolism”, “metabolic homeostasis”, “cardiometabolic risk”, “cardiometabolic markers”, and “polycystic ovary syndrome” or “PCOS” in conjunction with “characteristics”, “prevalence”, “diagnosis”, “guidelines”, “phenotypes”, “metabolism”, “metabolic homeostasis”, “hyperinsulinemia”, “insulin resistance”, “cardiometabolic risk”, “cardiometabolic markers”, “prolactin”, “hyperprolactinemia”, “gonadotropin-releasing hormone”, “luteinizing hormone”, “hyperandrogenemia”, “adiponectin”, “dopamine agonist”, “bromocriptine”, and “cabergoline”. We included articles published in English. 

## 2. The Emerging Role of Prolactin in Metabolic Homeostasis

### 2.1. Prolactin Synthesis and Regulation

PRL is a polypeptide hormone primarily produced by lactotrophs in the anterior pituitary gland [23,28]. It is secreted in pulses that follow a circadian rhythm [29,30]. Its production also occurs at extra pituitary sites, including various reproductive, immune, neural, and cutaneous tissues and sites such as adipose tissue (AT) [31,32]. Due to its mode of action, it can be classified as a circulating hormone and an autocrine and paracrine factor [25]. PRL represents the sole pituitary hormone for which release from the pituitary gland is controlled by the hypothalamic inhibitory tone. The most important inhibitor under physiological conditions is dopamine, secreted from the tuberoinfundibular system (TIDA) and acting via dopamine receptor D_2_. PRL also exerts a negative feedback function by promoting dopamine release [23,24,33]. On the other hand, the secretion of PRL is stimulated by thyrotropin-releasing hormone (TRH), estrogen, epidermal growth factor (EGF), vasoactive intestinal peptide (VIP), and dopamine receptor antagonists. In addition, a variety of physiological (intense exercise, acute stress, sleep, sexual arousal, etc.) and pathological conditions (prolactinomas, hypothyroidism, hepatic dysfunction, PCOS, etc.) can cause lactotrophic cells to increase their PRL secretion, possibly leading to HPRL [5,26,34]. Even though PRL is most commonly recognized for its effects on gonadal function, reproduction, and lactation, its transmembrane receptor (PRLR) is widely spread throughout the body. Therefore, PRL affects various tissues, making it a highly versatile and multifaceted hormone [23,25,35]. 

### 2.2. The Effects of Prolactin on Metabolism

An expanding body of clinical evidence suggests the involvement of PRL in metabolic homeostasis, encompassing aspects such as body weight regulation and functions within AT and the pancreas. It is also indicated that PRL’s effect on metabolic homeostasis largely depends on its circulating concentration [26,27,36]. In contrast to low and very high PRL levels, which have deleterious metabolic consequences, a specific range of PRL values benefits the metabolism. While the conventional classification of PRL levels considers normoprolactinemia as the value of PRL between 1 and 25 μg/L and everything above that as HPRL, this new specific range of values, considered beneficial for metabolic homeostasis, includes not only certain levels within the conventional physiological range (usually 7 to 25 μg/L) but also levels above the conventional hyperprolactinemic threshold (25 to 100 μg/L). This set of PRL values (25 to 100 μg/L) has now been defined as homeostatic functionally increased transient prolactinemia (HomeoFIT-PRL), as it refers to instances where PRL levels rise in reaction to either physiological or pathological stimuli, ultimately contributing to the maintenance of metabolic homeostasis [26,27,33]. 

Excessive levels of PRL (>100 µg/L) significantly affect metabolism. Regardless of the underlying cause, these elevated levels can influence central appetite regulation mechanisms, leading to hyperphagia and increased food intake. They are strongly linked to various metabolic alterations, including obesity, IR, dyslipidemia, non-alcoholic fatty liver disease (NAFLD), and endothelial dysfunction [33,37]. Notably, the physiological HPRL that occurs during pregnancy and lactation, reaching levels exceeding 100 µg/L, is a homeorhetic response and is not detrimental [26].

Interestingly, deficient levels of PRL (≤7 μg/L) have been associated with a similar degree of impairment of metabolic homeostasis [33,37]. Low PRL levels are associated with a higher prevalence of type 2 diabetes (T2D), IR, glucose intolerance, MS, AT dysfunction, ß-cell dysfunction, NAFLD, and cardiovascular events in humans [27,36].

In contrast, moderately high PRL levels correlate with metabolic protection [27,36]. 

Macotela et al. presented the concept of moderately elevated PRL levels as a part of a homeorhetic response to either physiological (e.g., pregnancy, lactation, stress) or pathological (e.g., obesity and MS) metabolic challenges, allowing a series of metabolic adaptations to deal with physio-pathological demand [27]. This response leads to a new physiological set point in a physiological challenge. In contrast, in a pathological challenge, the response leads to a milder disease or protection from the disease risk. Conversely, patients experiencing a metabolic challenge, such as obesity, who are unable to respond by increasing PRL levels are more prone to suffer from metabolic alterations than those able to upregulate their PRL levels. However, there remains a need to comprehend the mechanisms responsible for the increase in PRL levels during challenged metabolic homeostasis and the factors preventing this increase in certain individuals [27].

In summary, PRL exerts an important role in metabolic homeostasis, and its overall impact depends on its serum concentration. Excessive and deficient levels have been shown to impact metabolism negatively. Therefore, maintaining levels within the normal range is crucial for ensuring metabolic health. It is believed that PRL can sense an individual’s metabolic status. In response to physiological and pathological challenges, its levels increase as part of an adaptive response, allowing the body to adjust to demands appropriately. However, a pressing need remains for a more profound understanding of the underlying mechanism governing PRL level fluctuations during these challenges.

### 2.3. Mechanisms Mediating the Metabolic Action of Prolactin

Circulating PRL affects metabolic homeostasis by regulating key enzymes and transporters associated with glucose and lipid metabolism in several target organs [25,26]. The advantageous metabolic impacts of PRL are achieved through its direct interaction with target tissues [26]. PRL functions by binding to cell-surface PRLRs, thereby triggering signaling pathways. The PRLR is a member of the type-I cytokine receptor family, predominantly relaying signals via the Janus kinase 2/signal transducer and activator of the transcription 5 (JAK2-STAT5) pathway [38]. PRLRs can be found in nearly all bodily tissues, including vital metabolic organs such as the pancreas, liver, hypothalamus, and AT. The mechanisms through which PRL exerts its effects on these organs have mainly been elucidated through preclinical research [24,25,27]. 

PRL generally supports the growth of pancreatic islets, prevents ß-cell apoptosis, and stimulates insulin secretion [26,27]. The mechanisms underlying these effects involve an upregulation in the synthesis of osteoprotegerin. This has been demonstrated to promote the replication of human and rodent ß-cells by suppressing the receptor activator NF-kB pathway, which acts as an inhibitor of ß-cell proliferation [39]. In rodent models, PRL increased survivin levels [40], enhanced the expression of transcription factor forkhead box M1 (Foxm1) and MAF bZIP transcription factor B (MafB), and increased cyclin activity and islet serotonin production via tryptophan hydroxylase 1 (Tph1), all promoting ß-cell proliferation [41,42]. In isolated human pancreatic islets, PRL inhibited apoptosis pathways [43]. In rodents, it enhanced glucose sensitivity through increased glucose transporter 2 and glucokinase expression [44,45,46]. The latter regulates the rate-limiting step in glucose metabolism. The mechanism of increased expression is STAT5-dependent and leads to increased insulin secretion [38].

PRL might play a part in the storage of lipids within the liver, as research has shown lower circulating PRL levels in individuals with NAFLD [47]. PRL inhibits the expression of the fatty acid transketolase (CD36), a key transporter of free fatty acid uptake in the liver [33,47]. Furthermore, the gene expression of hepatic PRLR was notably diminished in patients with NAFLD, and this reduction showed a negative correlation with CD36 gene expression [47]. PRL decreased the expression of stearoyl-CoA desaturase 1 (SCD1) in animal models and multiple hepatic cell lines [48]. SCD1 is a rate-limiting enzyme for the biosynthesis of monounsaturated fatty acids that serve as substrates for de novo lipogenesis, thereby increasing the accumulation of triglycerides in the liver [49]. In mice, the insulin-sensitizing effects of PRL were mediated by the activation of STAT5 downstream of the PRLR [50]. PRLR interacts with insulin receptor substrate 1 (IRS1) and promotes the phosphorylation of protein kinase B (AKT), two key members of the insulin signaling pathway [50,51,52].

In a mouse model [53], research demonstrated that the overexpression of PRLRs in the hypothalamus improved hepatic insulin sensitivity, whereas inhibiting it had the opposite outcome. Moreover, this study identified a new central pathway that regulates hepatic insulin sensitivity, mediated by hypothalamic PRLR/STAT5 signaling and the vagus nerve. Additionally, it was observed that heightened PRLR expression in the hypothalamus promoted overall body insulin sensitivity [26,53].

In AT, PRL regulates lipid metabolism and promotes adipogenesis [26,27]. PRL diminished lipid absorption by lowering lipoprotein lipase activity via STAT5 in human fat [54]. Additionally, it impeded lipolysis in both rat and human AT [55], ultimately reducing adipocyte size. PRL promoted adipocyte differentiation in NIH-3T3 and 3T3-L1 adipocyte cell lines. This effect was mediated by the stimulation of STAT5 activation, along with the activation of adipogenic transcription factors, including CCAAT/enhancer-binding protein beta (CEBP/b) and peroxisome proliferator-activated receptor gamma (PPARg) [56,57]. In obese rats [58], PRL treatment induced adipocyte hyperplasia and decreased hypertrophy in visceral adipocytes. This effect was brought about by an upregulation in the expression of PPARg and the spliced variant of X-box-binding protein-1 (Xbp1s), both of which play roles in promoting adipogenesis and improving insulin sensitivity [58]. Hyperplasia is generally considered a relatively benign and possibly even protective mechanism for expanding AT. On the other hand, inadequate adipogenesis and excessive enlargement of fat cells (hypertrophy) are associated with dysfunction in AT and can contribute to MS [58]. 

These mechanisms may ultimately contribute to metabolic disorders in the presence of low PRL levels [36,59]. 

Research data by Ruiz-Herrera et al. implied that PRL at relatively high concentrations but within a physiological range found in female reproductive states may protect against metabolic dysfunction by increasing circulating adiponectin [58], a hormone secreted by adipocytes known for regulating glucose levels, lipid metabolism, and insulin sensitivity [60]. Moreover, a clinical study has shown a positive relationship between circulating PRL levels and serum adiponectin levels in normoprolactinemic women diagnosed with PCOS [35]. 

It is also worth noting that PRL not only exerts its effects on adipocytes but is also synthesized within human AT, potentially functioning as an autocrine or paracrine factor that plays a role in adipogenesis and the suppression of lipolysis [35,58]. Additionally, through the utilization of AT biopsies, the findings from the study mentioned above have suggested that both systemic PRL and PRL produced locally by the AT promote insulin sensitivity and AT metabolic homeostasis in humans [58]. 

Since much of our current knowledge on PRL has been obtained from studies on animal models, primarily rodents, translation to humans is limited. Animal studies have been a crucial avenue for studying and gaining insights into the human condition for centuries. While nearly all animal species share biological and pathobiological similarities with humans to some extent, they also possess distinct phenotypic characteristics. Acknowledging these unique traits in both normal and abnormal biology is paramount for comprehending research outcomes and grasping the implications of applying these models to the human context [61]. Notably, although some features of PRL and its actions are similar among rodents (mice and rats) and humans, there is a sufficient disparity in the control of the production, distribution, and physiological functions of PRL among these species [62]. Moreover, certain biological distinctions in conventional animal models are iatrogenic and are induced by our methods of care and study design [61]. Acknowledging that insights from laboratory animals are crucial for comprehending PRL’s role in human health and pathology is imperative. Nevertheless, considering PRL’s adaptable and versatile nature, any extrapolation from rodents to humans should be done carefully and thoughtfully [62].

To summarize, PRL influences metabolism directly by interacting with crucial target tissues, including the pancreas, liver, hypothalamus, and AT. These interactions are mediated through specific receptors, primarily utilizing the JAK2-STAT5 signaling pathway. PRL stimulates the proliferation of ß-cells and enhances insulin secretion while also preventing ß-cell apoptosis. In the liver, it plays a role in regulating lipid metabolism and liver insulin sensitivity. Within AT, PRL promotes metabolic fitness and encourages the formation of new adipocytes. Additionally, through its effects on the hypothalamus, PRL enhances insulin sensitivity. A better understanding of the underlying mechanisms of PRL’s impact on metabolism in pathological metabolic states would potentially lead to more precise individualized regulation of this hormone, ultimately leading to better health outcomes. There is a need for more comprehensive and robust data concerning PRL in clinical settings. This is essential in order to draw more definitive conclusions regarding the clinical implications of PRL in human physiology and health.

## 3. Prolactin and PCOS

### 3.1. Hyperprolactinemia and PCOS

PCOS is categorized as a potential etiology of HPRL [63]. Since PCOS and HPRL are among the most prevalent endocrine disorders in women of reproductive age, it is not uncommon for women undergoing evaluation for menstrual irregularities to be diagnosed with both conditions simultaneously. The connection and potential underlying mechanisms linking these two conditions have been of interest since the 1950s, but the available data in the literature remains ambiguous. The reported prevalence of HPRL in women with PCOS varies significantly, with figures ranging from 3% to as high as 67%. However, most of these studies were conducted before the first diagnostic criteria were published in 1990 by the National Health Institute (NIH). Since the diagnosis was established using the consensus criteria, these results have been more homogenous [64]. Delcour et al. have created a visual depiction illustrating the average occurrence of HPRL in women with PCOS. Their representation indicates a higher mean prevalence in studies before 2000, employing diverse criteria, compared to later studies utilizing the Rotterdam criteria—28% versus 11.9%, respectively [64]. Since then, more studies have reported the prevalence of HPRL in their groups of PCOS patients, ranging from 11.6% to 37% [65,66,67,68,69].

Nonetheless, data from many of these studies did not seem to confirm the presence of idiopathic or PCOS-related HPRL in women with PCOS when a rigorous etiological investigation was conducted. These studies have shown that in most cases, there is a classical cause for HPRL (e.g., prolactinomas, hyperprolactinemic drug, macroprolactin) in PCOS women who present with elevated serum PRL levels [13,66,67,70,71]. For example, in a recent study by Davoudi et al. [66], which included 330 patients diagnosed with PCOS based on Rotterdam criteria, normal PRL levels were detected in 63% and HPRL was diagnosed in 37% of patients. The average PRL levels were 16.93 ± 4.81 μg/L for those with normal PRL levels and 48.42 ± 5.44 μg/L for those with HPRL. However, after excluding other known causes of HPRL, the prevalence of idiopathic or PCOS-related HPRL was 5%, with the mean value of PRL being 44.56 ± 8.85 μg/L. Notably, they could not exclude the presence of microadenoma because of the limited resolution of pituitary MR imaging (MRI) [66]. In a similar study [67], the overall prevalence of HPRL was 11.7%. Of all the 179 patients in the PCOS cohort, 3.4% had unexplained HPRL despite complete investigation, including PRL chromatography, pituitary MRI, exclusion of hypothyroidism, pregnancy, and HPRL drugs. These idiopathic cases displayed slight elevations in PRL, with an average PRL level of 33.4 ± 9.3 μg/L. Moreover, all of them had a marked response (i.e., >300% from baseline PRL) to the metoclopramide test, contradicting the likelihood of picoprolactinomas being present (i.e., adenomas typically smaller than 3 mm, often not visible on pituitary MRI scans). Nevertheless, whether a subtle interplay between PCOS pathophysiology and the mechanisms of PRL secretion is operational in these instances remains a subject requiring further investigation [67]. Of note, both studies considered patients hyperprolactinemic if they had at least two PRL measurements above the normal upper limit [66,67]. 

Moreover, investigators using serial serum sampling instead of single PRL measurements have excluded transient PRL elevations (e.g., an elevated PRL resulting from acute stress) and have shown a less frequent association between PCOS and HPRL [72,73]. There was a study conducted to investigate the benefits of assessing circadian profiles compared to single PRL sampling in PCOS patients [74], in which blood samples were collected from the cubital vein at three-hour intervals throughout the day using a previously inserted cannula. When measured through daily profiles, their data showed no connection between elevated PRL levels and PCOS. Additionally, their findings indicated that HPRL does not appear to occur more frequently in women with PCOS than in healthy individuals, suggesting that PCOS and HPRL are separate clinical conditions [74].

Nevertheless, there is still insufficient data to be conclusive on the subject, and a rigorous study on a larger cohort of patients with PCOS is needed to confirm these findings [64]; consequently, excluding other causes of HPRL is warranted in all patients with increased PRL levels.

### 3.2. Serum Prolactin Levels in PCOS Women Compared to Healthy Controls

Women with PCOS are reported to exhibit modified PRL levels compared to those without the syndrome. Over the years, various research groups explored circulating PRL levels in women with PCOS compared to their non-PCOS controls. However, this issue still demands further high-quality research efforts, as studies to date report inconsistent results with inconsistent study designs, even after the adjustment for potential factors that could influence the outcome (e.g., age and BMI). The first and only published meta-analysis to date [5] exploring the difference in PRL levels in women with PCOS and their non-PCOS controls included 32 studies. These studies focused on patients diagnosed using Rotterdam criteria and excluded other potential causes of elevated PRL levels [5]. Their findings demonstrated significantly higher PRL levels among individuals with PCOS than those without the condition. The analysis included a total of 22,288 participants, consisting of 8551 individuals with PCOS and 13,737 individuals without the condition. The calculated weighted mean difference (WMD) was 1.01 μg/L, with a 95% confidence interval (CI) spanning from 0.04 to 1.98 (*p* = 0.040). Further meta-regression analysis revealed that age, BMI, and the participants’ continent of origin did not substantially influence PRL levels in either the patient or the control group. They also conducted literature research, showing the effect that using different diagnostic criteria has on the result of such studies, as studies performed with patients diagnosed with NIH or Androgen Excess Society (AES) criteria showed no significant difference in PRL levels between the two groups [5]. However, some individual studies, even within this meta-analysis, have found statistically lower PRL levels in women with PCOS than their non-PCOS peers [15,75,76,77]. Of note, these studies included only normoprolactinemic PCOS participants. 

Discrepancies between findings could, at least in part, be explained by the following limitations [5,15,75,76,77]. First, it is important to note that most studies to date had small sample sizes, reducing their statistical power. They had heterogeneous study populations, with some research groups excluding women with HPRL and some only including infertile women. There was a lack of explanation of methods used to exclude other possible etiologies of HPRL. Time, as well as the blood sample collection to detect PRL concentration, could affect the results. On one hand, PRL secretion displays diurnal variation with a nocturnal peak in the late-night/early morning hours. On the other hand, stress is a known cause of transient HPRL. Since venipuncture is considered a source of stress for the patient, information on whether a one-time fasting sample was used in comparison to serial sampling, which should be the standard method for those studies, is essential [78]. There was also a variety of assay methods used. Furthermore, the considerable diversity observed in traits among women diagnosed with PCOS might contribute to the differences in PRL levels. The absence of detailed descriptions of each study group’s patient characteristics and phenotypes could contribute to inconsistent findings [22].

The precise physiological mechanism responsible for the alterations in PRL levels observed in women with PCOS remains the subject of ongoing research. Several hypotheses have been proposed to justify the elevated PRL levels. A commonly proposed theory suggests a mutual dysfunction in the hypothalamic–pituitary axis, which could explain both conditions concurrently. Women diagnosed with PCOS reportedly have higher gonadotropin-releasing hormone (GnRH)/LH pulse frequency, which might account for increased PRL levels [64,79]. It has been hypothesized that the irregular secretion of gonadotropins in PCOS could stem from disruptions within the underlying neuroendocrine process that governs the release of GnRH [80]. Persistently high GnRH pulse frequency favors LH production over FSH production. Consequently, women with PCOS exhibit exaggerated LH production and relative FSH deficiency [81]. The response of ovarian granulosa cells to a decreased frequency of FSH release results in a disruption in the selection of dominant follicles. This leads to the stagnation of follicle development, culminating in the characteristic polycystic appearance observed during ultrasound examinations, often associated with infertility. On the other hand, heightened LH levels stimulate theca cells within the ovary to overproduce androgens, contributing to hyperandrogenemia. The elevated serum androgen levels in PCOS also prompt the peripheral synthesis of estrogen [82]. However, mechanisms underlying rapid pulsatile GnRH secretion remain poorly understood [81]. It seems that increased GnRH pulsatility in PCOS is mediated by reduced sensitivity to negative feedback from sex steroids (progesterone and estradiol), which, at least in part, is due to elevated androgen levels [83]. Moreover, dopamine is a major suppressor of GnRH release, and many studies suggest the role of reduced dopaminergic tone in increased LH release in PCOS. In addition to its influence on GnRH/LH, dopamine inhibits PRL release [79]. Some older studies show a synchronization between the PRL and LH secretion peaks in women with PCOS [72,84,85]. It has thus been hypothesized that the high levels of PRL found in women with PCOS could be secondary to a decrease in dopaminergic tone, which is linked to increased LH release [86,87,88]. The causality of the association between higher levels of PRL and PCOS is still unclear; higher levels of PRL could inhibit ovulation and lead to polycystic ovarian morphology [5]. A recent study concluded that in PCOS women aged ≤ 35, the upper reference limit of PRL is approximately 1.5 higher than in controls [68]. The authors of this study have shown that PCOS women with higher levels of LH are more likely to show a mild increase in PRL levels. However, it does not seem that this level of PRL elevation could be a causative factor for irregular cycles or amenorrhea in women with PCOS because amenorrhea/oligomenorrhea occurs at PRL levels of more than 50 μg/L, while PRL levels ranging from 25 to 50 μg/L do not usually cause notable changes in menstrual cycles, though they may decrease overall fertility [68,89]. What is intriguing is that PRL actually has an inhibitory effect on the pulsatile release of GnRH, resulting in decreased LH levels. However, it appears that the relatively mild elevation of PRL seen in PCOS is insufficient to counteract the GnRH pulsatility, in keeping with the dominance of LH secretion [68].

Another proposed mechanism is founded on the association of PCOS with hyperandrogenemia and relatively high estrogen levels, which could stimulate PRL secretion [34]. The latter is supported by findings that show a positive correlation between PRL and estradiol/testosterone levels [15]. In light of discoveries about the protective role of medium and moderately elevated PRL levels, we could also hypothesize that elevated PRL levels found in some patients with PCOS could be due to homeorhetic responses to pathological metabolic challenges found in this syndrome (e.g., obesity, IR, hyperlipidemia) [26,27].

However, none of the aforementioned hypotheses explains lower PRL levels found in some women with PCOS compared to their non-PCOS controls. Studies researching the pathophysiology of obesity have shown a presence of reduced hypothalamic dopaminergic action in obese patients [29,90,91]. Since dopamine normally tonically inhibits PRL secretion, one would anticipate elevated PRL levels. Paradoxically, the opposite is observed, with reduced PRL levels observed in some cases of obesity and metabolic disease, suggesting a hypothalamic–pituitary dysfunction might be to blame for the alterations in PRL levels [26,29]. Moreover, a study by Albu et al. focused on the relationship between the serum PRL level and AT in PCOS [35]. While some authors have suggested that locally produced PRL acts mainly as an autocrine or paracrine factor [25], the data from this study proposed a significant contribution by adipocyte-derived PRL to circulating PRL [35]. Their findings suggested that in PCOS patients, serum PRL levels are influenced by both AT quantity and function [35]. Importantly, AT dysfunction is implicated as a key feature in PCOS, and both lean and obese women with the syndrome have aberrant AT morphology [92]. The authors showed an inverse association between the serum PRL level and visceral adiposity index (VAI) [35], which was shown to be correlated with the visceral fat area (measured with computed tomography) [93]. This finding contradicts data presented in a different study, wherein it was demonstrated that premenopausal women with visceral obesity exhibit greater basal and pulsatile secretion of PRL compared to lean control subjects [94]. However, they concluded that this could suggest that the relationship between visceral adiposity and PRL could differ according to the study population [35]. In contrast with an earlier study [95], they found a positive association between PRL and adiponectin, which is decreased in the presence of AT dysfunction [96]. VAI was also demonstrated to indicate adiposity dysfunction [97]. Additionally, if PRL elevation in PCOS indicates a homeorhetic response, low PRL levels in patients with PCOS could indicate an inability to upregulate PRL secretion, meaning their concentrations stay low. Consequently, these patients are more prone to suffer the consequences of those metabolic alterations [26,27].

Nonetheless, considering the existing data, it is essential to conduct additional research into the mechanism underlying the changes in PRL levels in PCOS.

### 3.3. Association between Serum PRL Levels and Markers of Metabolic Risk in PCOS

Many women with PCOS are obese or overweight and exhibit impaired insulin metabolism, with IR and hyperinsulinemia being largely present in lean women with PCOS as well. IR, together with ß-cell dysfunction, increases the risk of developing other metabolic abnormalities such as T2D, hypertension, dyslipidemia, and cardiovascular disease [2,98]. Obesity and IR are metabolic stimuli that could stimulate homeorhetic response and elevate PRL levels to a specific range that promotes metabolic homeostasis [26,27].

**Figure 1:**
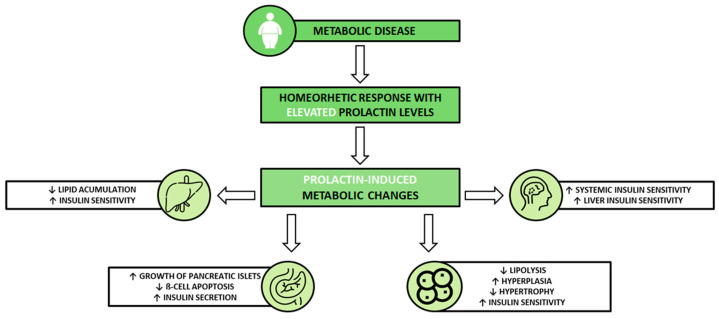
Potential benefits of homeorhetic response in women diagnosed with polycystic ovary syndrome (PCOS). Metabolic disease triggers an increase in prolactin (PRL) levels. PRL subsequently binds to cell-surface PRL receptors in target tissues, including the pancreas, liver, hypothalamus, and adipose tissue (AT), initiating signaling pathways. This sequence of events leads to metabolically advantageous effects of PRL in these tissues, ultimately contributing to a favorable metabolic profile. PRL stimulates the growth of pancreatic islets and enhances insulin secretion while preventing ß-cell apoptosis. In the liver, it reduces lipid accumulation and promotes insulin sensitivity. Within AT, PRL inhibits lipolysis, stimulates insulin sensitivity, and encourages adipocyte hyperplasia while reducing adipocyte hypertrophy. Additionally, through its effects on the hypothalamus, PRL enhances insulin sensitivity [26,27].

Figure 1 illustrates the potential shift in PRL levels as a component of a broader homeorhetic response to a metabolic disease, resulting in advantageous impacts of PRL on specific tissues. This may ultimately contribute to a compensatory response to improve the metabolic profile in women diagnosed with PCOS.

Over the past years, few studies have explored the connection between the serum PRL levels and metabolic profiles of women with PCOS. All these studies were conducted on normoprolactinemic premenopausal women with PCOS who were diagnosed using Rotterdam criteria [15,35,76,77].

The first such study was conducted in 2014 [15]. It showed an inverse correlation between PRL levels and waist circumference (WC), triglycerides (TG), total cholesterol (TC), and low-density lipoprotein cholesterol (LDL-C), but a positive association between PRL and high-density lipoprotein cholesterol (HDL-C) [15]. Furthermore, the significant inverse association between PRL and LDL-C was independent of smoking status, BMI, and age, all of which can affect PRL levels. When PCOS patients were divided into two groups according to their median PRL level of 7 μg/L, which also represents the lower limit for the range of PRL levels that are considered beneficial for metabolic homeostasis, they showed that PRL levels below that cut-off are associated with unfavorable lipid profiles. However, smoking prevalence and age were statistically higher in the group with lower PRL [15]. 

In 2016, a similar study was published, with additional data on the association between serum PRL levels and AT quantity and function in PCOS patients [35]. There was an inverse correlation between serum PRL and all adiposity markers (body mass index (BMI), waist-hip ratio (WHR), visceral adiposity index (VAI), WC), suggesting a complex bidirectional relationship between PRL and AT, with PRL influencing adipogenesis and adipocyte function (low PRL usually being associated with adipocyte hypertrophy in visceral but not subcutaneous fat), and, in turn, with AT being able to produce PRL [59]. All results remained statistically significant even after the adjustment for age and BMI. Additionally, when determining whether the serum PRL level is an independent predictor of metabolic abnormalities in PCOS patients, they discovered associations of low serum PRL with an unfavorable metabolic profile, including the presence of MS. Results showed an inverse association between PRL and the homeostasis model of assessment of insulin resistance (HOMA-IR); however, the statistical significance was lost after adjustment for age and BMI/WC. There was also a negative correlation between PRL levels and fasting glucose in patients with normal glucose homeostasis but not in patients with glucose metabolism abnormalities (T2D, impaired fasting glucose, or impaired glucose tolerance) [35]. 

Two other studies were conducted by the same author, studying a population of infertile Chinese women [76,77]. To exclude the influence of age, they divided patients into different age groups. Their results showed a significantly lower PRL in women with PCOS than infertile controls in each age group, even after adjusting for BMI. The two studies showed, similarly to previous ones [15,35], that low PRL may be a significant cause of metabolic risk. In the first study [76] they, consistent with a study by Albu et al. [35], found inverse correlations between serum PRL and BMI, as well as TG, TC, and LDL-C in patients with PCOS, and showed that even after the adjustment for BMI, levels of these markers are still significantly higher in PCOS than in non-PCOS infertile patients. In contrast to earlier research findings, they observed a positive relationship between PRL and fasting glucose levels. However, this connection did not reach statistical significance in their subsequent study. Their analysis showed that PRL is negatively correlated with aspartate transaminase (AST), alanine transaminase (ALT), gamma-glutamyl transferase (GGT), and alkaline phosphatase (ALP), suggesting that low PRL may damage liver cells [76]. The second of the two studies [77], however, focused more on investigating the association of serum PRL with IR and ß-cell dysfunction in infertile PCOS patients. They observed that serum PRL levels in women with PCOS were inversely associated with WC, hip circumference (HC), fasting insulin (FINS), HOMA-IR, and the homeostasis model assessment of ß-cell function (HOMA-ß). Consequently, they deduced that low PRL levels within the normal range may be associated with increased WC and HC and a higher risk for IR. Moreover, they proposed that serum PRL levels in infertile women with PCOS might be a predictor for IR and ß-cell dysfunction. Consistent with other studies, they found a negative correlation of PRL levels with TG, a positive correlation with HDL-C, and an inverse association with ALT [77]. After adjusting for age and BMI, the analysis revealed a negative association between PRL and FINS, HOMA-IR, and HOMA-ß [76,77].

Table 1 provides an overview of the main outcomes of the four clinical studies discussed in detail above, which assessed the association of serum PRL levels and metabolic markers in patients with PCOS.

These studies have provided limited but valuable insights into the potentially beneficial metabolic effects of PRL in individuals with PCOS, as they show a positive correlation between a favorable metabolic profile and serum PRL levels [15,35,76,77]. They associate lower PRL levels with metabolic risks in patients with PCOS and subsequently label low PRL as a metabolic risk factor. Because all of the patients with PCOS in these studies had PRL levels below the upper limit of normal, key findings support the assumption that even serum PRL levels within the physiological range are associated with changes in glucose and lipid metabolism in this disorder. Nevertheless, it is crucial to emphasize that all of these studies were cross-sectional, and it is imperative to conduct longitudinal studies to gain a deeper understanding of how PRL influences metabolic outcomes. Although alluding to it, the questions of elevated PRL levels as a mechanism to counteract metabolic alterations in PCOS and the beneficial effects of medium and moderately elevated PRL levels above the conventional physiological range in PCOS remain a subject of future studies. Moreover, the presently acknowledged four phenotypes of PCOS per Rotterdam criteria do not directly consider metabolic dysfunction underlying PCOS [22]. Therefore, establishing new phenotypes among PCOS patients that would also consider patients’ metabolic status remains a promising future research perspective.

## 4. Role of Dopamine Agonists

DAs have broad clinical applications. They are crucial in managing conditions such as Parkinson’s disease and restless leg syndrome. Certain DAs, such as fenoldopam, can be used in critical cases of hypertensive emergencies. Additionally, DAs can be considered in more severe cases of neuroleptic malignant syndrome. Their effectiveness in regulating PRL levels makes them the first-choice treatment for patients with HPRL [99,100].

According to etiopathogenesis, two distinct types of HPRL exist—organic and functional. The main causes of organic HPRL are typically prolactinomas or damage to dopaminergic neurons in the hypothalamus or masses that press on the pituitary stalk. On the other hand, pathological functional HPRL arises from various endocrine disorders, such as primary hypothyroidism and primary adrenocortical insufficiency [101]. Generally, HPRL found in certain patients with PCOS is also regarded as functional [65].

As discussed, dopamine released by the hypothalamus is the primary physiological PRL inhibitory factor acting on the D_2_ dopamine receptor on the lactotrophs’ surface membrane. DAs work by mimicking the action of dopamine within the pituitary. They enhance dopamine production and directly impact dopamine receptors, thereby inhibiting the release of PRL [100]. Most individuals diagnosed with either micro- or macroprolactinomas can achieve successful treatment using DAs. This treatment not only restores normal PRL secretion and gonadal function but also substantially reduces tumor size. Cabergoline (CAB) and bromocriptine (BRC) represent the most commonly prescribed DAs. Although both express a high affinity for dopamine D_2_ receptors in the pituitary, CAB is the preferred option due to its favorable characteristics. These include a longer plasma half-life, enabling less frequent administration (once or twice weekly) in contrast to BRC, which may require multiple daily doses. Additionally, CAB demonstrates higher effectiveness and improved tolerability [30,102]. Less frequent dosing and improved tolerability may lead to better patient compliance with CAB than BRC [102]. Furthermore, it has been proven effective in patients who are resistant or minimally responsive to BRC [103].

While DAs have shown effectiveness in managing PRL excess and present with a low side-effect profile, it is essential to remain vigilant about potential adverse reactions [104]. The primary side effects frequently associated with DAs encompass nausea, orthostatic hypotension, and headaches. Rarely, valvulopathy can occur with high doses of DAs. Additionally, there have been documented instances of psychosocial symptoms such as mania, anxiety, and depression. Furthermore, DAs sometimes lead to impulse control disorders, manifesting as excessive shopping, gambling, and heightened sexual desire [105,106,107,108].

Research has demonstrated that using BRC [109,110] and CAB [111] as monotherapies has significantly decreased PRL levels in individuals affected by both HPRL and PCOS concurrently. More recent studies have explored the impact of CAB in combination with metformin on PRL levels and have shown a significant decrease in PRL and a higher rate of cycle regulation following this intervention [112,113]. Furthermore, when administered to women with PCOS, BRC has shown a noteworthy reduction in LH levels in patients with both normal and elevated PRL levels [110,114]. In certain instances, the use of BRC has effectively restored regular ovarian cycles [114]. Additionally, evidence suggests that CAB can reduce PRL concentrations, normalize androgen levels, and ameliorate menstrual irregularities in women diagnosed with PCOS without modifying LH secretion [115]. However, similarly to an earlier study done on normoprolactinemic women with PCOS [116], a study conducted on PCOS women resistant to clomiphene who had normal PRL levels before beginning BRC treatment found that the primary significant effect of long-term BRC therapy in these patients was a reduction in serum PRL concentrations. Nonetheless, no significant differences were observed in ovulation, pregnancy rates, or serum levels of FSH, LH, DHEAS, and progesterone between the group receiving BRC and the placebo group after treatment [117]. Another study comparing acute effects of BRC on PRL levels in patients with hyperprolactinemic and normoprolactinemic PCOS demonstrated an inhibitory effect of DAs on LH levels only in PCOS women with concurrent HPRL, suggesting that a relative endogenous dopamine deficiency may be, in part, the cause of hypersecretion of PRL and LH in patients with hyperprolactinemic PCOS [118]. In conclusion, while certain studies highlight the influence of DAs on the hormonal profiles of women with PCOS, such as the reduction in LH and PRL levels and the restoration of regular ovarian cycles, others suggest that their benefits may be limited.

Notably, several studies have demonstrated that drugs aimed at reducing PRL, such as DA, positively impact metabolic profiles [37]. The observation may initially seem contradictory to the hypothesis that an optimal range of PRL levels exists for maintaining metabolic homeostasis [26]. However, it is proposed that dopamine and elevated PRL levels might enhance metabolic fitness through distinct mechanisms [37,119]. Furthermore, maintaining PRL levels within the normal range seems crucial for metabolic homeostasis. Therefore, it might be advisable to target for PRL levels within the mid-normal range in patients with high PRL levels receiving DAs. This approach may help avoid potential adverse metabolic consequences associated with excessively low PRL [33]. In future research, randomized controlled studies should also explore the utilization of tricyclic antidepressants with an already low degree of evidence for their potential to modulate PRL levels.

## 5. Conclusions

The available data on serum PRL levels in women with PCOS present conflicting findings. Recent studies have not consistently supported the existence of PCOS-related HPRL. When using a comprehensive diagnostic approach, which involves investigations into potential causes of elevated PRL levels such as PRL chromatography, pituitary MRI, and ruling out other factors such as hypothyroidism, pregnancy, and the use of drugs that may elevate PRL, a significant portion of PCOS patients with elevated PRL levels were found to have other concurrent factors responsible for the HPRL. These factors include conditions such as prolactinomas, the use of hyperprolactinemic drugs, and the presence of macroprolactin. Studies examining the occurrence of HPRL in women with PCOS found that idiopathic HPRL was present in less than 5% of the participants. Future research is needed to uncover the precise mechanisms that explain the connection between PRL levels and PCOS.

Notably, extremely high or deficient PRL levels can lead to detrimental metabolic alterations, such as obesity, MS, and disruptions in glycemic and lipid profiles. Conversely, mid-normal and moderately high levels of PRL (ranging from 7 to 100 μg/L) may support metabolic homeostasis when facing various metabolic challenges. Despite increasing awareness of its dual influence, limited research has explored how PRL levels affect the metabolism of women with PCOS. 

Studies on normoprolactinemic PCOS patients suggest that PRL levels within the upper physiological range are associated with favorable metabolic profiles. On the other hand, low PRL levels indicate a metabolic risk in these individuals. All these studies were cross-sectional, and it is imperative to conduct long-term longitudinal studies to gain a more comprehensive understanding of how PRL affects the metabolic health of women with PCOS. Longitudinal studies should investigate the correlation between PRL levels and markers of metabolic risk that were already studied in the above-mentioned cross-sectional studies (i.e., adiposity markers (BMI, WHR, VAI, WC), TC, TG, LDL-C, HDL-C, fasting glucose, FINS, HOMA-IR, HOMA-ß), as well as the correlation with oral glucose tolerance test (OGTT) results and body composition, in particular with visceral adiposity. Furthermore, future research should explore interventions with the potential to modify prolactin levels and their effects on the reproductive and metabolic health of women with PCOS. Randomized controlled studies should further investigate the effects of DAs in specific PCOS phenotypes as well as explore unaddressed possibilities, such as the use of tricyclic antidepressants with an already low degree of evidence for their potential to modulate PRL levels. 

Overall, the existing literature on PRL and its role in PCOS sparks numerous thought-provoking questions, emphasizing the pressing need for additional in-depth research in this area. This will advance our understanding of PCOS and pave the way for more targeted and effective interventions for individuals affected by this condition.

## Figures and Tables

**Table 1:** Main outcomes of studies evaluating the association of serum PRL and metabolic markers.

Study	Sample Size, Participants	Age (Years)	BMI of Participants (kg/m^2^)	PRL (μg/L)	Main Outcomes
Glintborg, 2014 [15]	1007, patients with PCOS	30 (23–36)	27.4 (23.2–33)	7 (5–10)	PRL was inversely associated with WC (*r* = −0.13, *p* < 0.05), TC (−0.13, *p* < 0.05), LDL-C (*r* = −0.15, *p* < 0.05), TG (*r* = −0.14, *p* < 0.05), and positively with HDL-C (*r* = 0.11, *p* < 0.05)
Albu, 2016 [35]	322, patients with PCOS	24 (IQR 7)	27 (IQR 11.8)	12.9 (IQR 8.25)	PRL was inversely associated with HOMA-IR (*r* = −0.185, *p* = 0.002), BMI (*r* = −0.194, *p* < 0.0001), WC (*r* = −0.217, *p* < 0.0001), and fasting glucose (*r* = −0.123, *p* = 0.037)
Yang, 2020 [76]	2052, infertile patients with PCOS	29.12 ± 0.63	22.8 ± 1.2	11.71 ± 1.92	PRL was inversely associated with BMI (*r* = −0.015, *p* < 0.01), TG (*r* = −0.067, *p* < 0.01), TC (*r* = −0.089, *p* < 0.01), LDL-C (*r* = −0.074, *p* < 0.01)
Yang, 2021 [77]	792, infertile patients with PCOS	29 (27–32.5)	23.73 (21.48–26.85)	11.08 (8.78–14.95)	PRL was inversely associated with BMI (*r* = −0.086, *p* < 0.05), WC (*r* = −0.302, *p* < 0.001), TG (*r* = −0.107, *p* < 0.05), FINS (*r* = −0.152, *p* < 0.001), HOMA-IR (*r* = −0.144, *p* < 0.001), HOMA-ß (*r* = −0.165, *p* < 0.001), and positively with HDL-C (*r* = 0.084, *p* < 0.05)

Legend: PRL = prolactin; PCOS = polycystic ovary syndrome, BMI = body mass index; WC = waist circumference; TG = triglyceride; TC = total cholesterol; LDL-C = low-density lipoprotein cholesterol; HDL-C = high-density lipoprotein cholesterol; HOMA-IR = homeostasis model assessment of insulin resistance; HOMA-ß = homeostasis model assessment of ß-cell function; FINS = fasting insulin. Data for continuous variables are expressed as mean ± standard deviation (SD) or median (interquartile range). Different reported measurement units of PRL in different studies were all converted to a single unit, μg/L.

## Data Availability

Not applicable.
